# Cow Dung Is a Novel Feedstock for Fibrinolytic Enzyme Production from Newly Isolated *Bacillus* sp. IND7 and Its Application in *In Vitro* Clot Lysis

**DOI:** 10.3389/fmicb.2016.00361

**Published:** 2016-03-29

**Authors:** Ponnuswamy Vijayaraghavan, Arumugaperumal Arun, Samuel Gnana Prakash Vincent, Mariadhas Valan Arasu, Naif Abdullah Al-Dhabi

**Affiliations:** ^1^International Centre for Nanobiotechnology, Centre for Marine Science and Technology, Manonmaniam Sundaranar UniversityRajakkamangalam, India; ^2^Department of Biotechnology, Kalasalingam UniversitySrivilliputtur, India; ^3^Department of Botany and Microbiology, Addiriyah Chair for Environmental Studies, College of Science, King Saud UniversityRiyadh, Saudi Arabia

**Keywords:** agroresidues, cow dung, *Bacillus* sp. IND7, solid-state fermentation, fibrinolytic enzyme, response surface methodology

## Abstract

Bacterial fibrinolytic enzymes find great applications to treat and prevent cardiovascular diseases. The novel fibrinolytic enzymes from food grade organisms are useful for thrombolytic therapy. This study reports fibrinolytic enzyme production by *Bacillus* sp. IND7 in solid-state fermentation (SSF). In this study, cow dung was used as the cheap substrate for the production of fibrinolytic enzyme. Enzyme production was primarily improved by optimizing the nutrient and physical factors by one-variable-at-a-time approach. A statistical method (two-level full factorial design) was applied to investigate the significant variables. Of the different variables, pH, starch, and beef extract significantly influenced on the production of fibrinolytic enzyme (*p* < 0.05). The optimum levels of these significant factors were further investigated using response surface methodology. The optimum conditions for enhanced fibrinolytic enzyme production were 1.23% (w/w) starch and 0.3% (w/w) beef extract with initial medium pH 9.0. Under the optimized conditions, cow dung substrate yielded 8,345 U/g substrate, and an overall 2.5-fold improvement in fibrinolytic enzyme production was achieved due to its optimization. This is the first report of fibrinolytic enzyme production using cow dung substrate from *Bacillus* sp. in SSF. The crude enzyme displayed potent activity on zymography and digested goat blood clot completely in *in vitro* condition.

## Introduction

Fibrinolytic enzyme is considered as the potent thrombolytic agent to treat and prevent cardiovascular diseases (CVDs) (Mine et al., 2005). The commercially available thrombolytic agents such as urokinase plasminogen activator and tissue plasminogen activator are generally safe but are very expensive. However, the bacterial streptokinase is a cheap thrombolytic agent but causes undesirable side effects such as bleeding complications (Banerjee et al., 2004). Hence, searching of a potent thrombolytic agent to prevent and treat CVDs continues. The fibrinolytic enzyme that was isolated from food-grade microorganisms attracted attention worldwide (Mine et al., 2005). In recent years, fibrinolytic enzyme has been screened and characterized from various sources including *Bacillus thuringiensis* IMB B-7324 (Matseliukh et al., 1999), *B. thuringiensis* IMV B-7324 (Nidialkova et al., 2012), *Streptomyces* sp. MCMB (Chitte et al., 2011), *Virgibacillus* sp. SK37 (Lapsongphon et al., 2013), *B. thuringiensis* IMV B-7324 (Nidialkova et al., 2013), *B. subtilis* RJAS19 (Kumar et al., 2014), *Proteus penneri* SP-20 (Jhample et al., 2015), and *Bacillus* sp. (Anh et al., 2015). In this study, fermented rice was used for screening of fibrinolytic enzyme-producing organisms.

Solid-state fermentation (SSF) is defined as the growth of microorganisms on solid materials for the production of biomolecules in the absence or near absence of free water (Pandey et al., 2000). SSF is a useful technique for utilization of low-cost agroresidues in large volumes in biosynthesis of enzymes and metabolites. The agroindustrial wastes such as pigeon pea (Johnvesly et al., 2002), green gram husk (Prakasham et al., 2006), potato peel (Mukherjee et al., 2008), *Jatropha curcas* seed cake (Mahanta et al., 2008), sesame oil cake (Rajendran and Thangavelu, 2013), and ground nut husk (Salihu et al., 2014) were recently used as the substrate for the production of hydrolytic enzymes. The ideal agro-wastes for enzyme production in SSF process mainly depend on the cost and the availability of the substrate material (Pandey et al., 2000). Hence, the search for a novel and inexpensive substrate for the production of fibrinolytic enzyme is a continuous process.

In SSF process, the solid materials provide nutrients to the growth of microorganisms. Hence, the selected solid substrate for any bioprocess should contain enough nutrients for the growth and the production of enzymes. Cow dung is one of such nutritive-rich feedstocks that was unexploited for the production of fibrinolytic enzymes by *Bacillus* sp. Cow dung contains ash, nitrogen, carbon, cellulose, hemicelluloses, magnesium, manganese, calcium, zinc, and trace elements (Fulhage, 2000). Cow dung manure is rich in carbon and nitrogen, which indicated that it could be a promising feedstock for the growth of microbes (Adegunloye et al., 2007). In recent years, cow dung was used as the substrate for the production of proteolytic enzymes from *Halomonas* sp. PV1 (Vijayaraghavan and Vincent, 2012) and *Bacillus* sp. (Vijayaraghavan et al., 2012) and fibrinolytic enzymes from *Shewanella* sp. IND20 (Vijayaraghavan and Vincent, 2015). Unlike other solid substrates, cow dung has high moisture-holding capacity, which was preferred by the bacterial species for their growth and production of biomolecules (Vijayaraghavan and Vincent, 2015). Although cow dung was utilized for the production of proteolytic enzymes from *Bacillus* sp., the production of fibrinolytic enzyme from the genus *Bacillus* using cow dung substrate has not yet been reported.

Statistical experimental design helps to identify the significant factor and to find the optimum concentration for enzyme production. The traditional one-factor-at-a-time strategy frequently fails to locate the optimum response in an enzyme bioprocess. Hence, designing of suitable culture medium for enhanced production of enzymes or biomolecules by mathematical model is critically important. Statistical tools such as Plackett–Burman (Dilipkumar et al., 2011), L_18_-orthogonal array (Mahajan et al., 2012), and fractional factorial design (Liu et al., 2005) were used to screen the variables. Response surface methodology (RSM) helps to locate the optimum concentration of these significant variables. It was widely used for the production of acid proteases (Siala et al., 2012), amylases (Abdel-Fattah et al., 2013), and fibrinolytic enzymes (Vijayaraghavan and Vincent, 2014). Considering the nutrient compositions, availability, and cheap cost, cow dung was used as the substrate for the production of fibrinolytic enzyme from the newly isolated *Bacillus* sp. IND7, and the process parameters were optimized. In addition, the *in vitro* clot lytic activity of this enzyme was evaluated.

## Materials and Methods

### Screening of Fibrinolytic Enzyme-Producing Bacterial Strains from Fermented Rice

#### Primary Screening

Cooked rice was used as the sample source to screen protease enzyme-producing bacterial strains. Rice was boiled in drinking water for 1 h approximately. After that, the boiled rice was allowed under aerobic fermentation at room temperature (30 ± 2°C) for 48–72 h. sampling was made twice in each experimental trial (after 48–72 h fermentation). This procedure was repeated five times. About 1.0 g of cooked rice was suspended in 99 ml of double-distilled water and serially diluted (10^-1^–10^-7^) using sterile double-distilled water and plated on skimmed milk agar plates (g/l; agar 15, skim milk 10, peptone 5, yeast extract 5, and NaCl 1.5). These plates were incubated at 37°C for 24–72 h. The protease-producing bacterial isolate shows a clear zone on skimmed milk plates. In each trial, one organism was selected and further screened (secondary screening) for fibrinolytic enzyme production.

#### Secondary Screening

The selected five protease-producing bacterial isolates were cultured in the liquid medium composed of casein 10, yeast extract 5, peptone 5, and NaCl 1.5 (g/l). The culture medium was sterilized at 121°C for 20 min. Then, a loopful culture of the selected isolates was individually inoculated. Fermentation was carried out in 250-ml Erlenmeyer flasks, and these were kept on an orbital shaker (175 rpm) for 48 h at 37°C. After 48 h, all cultures were centrifuged at 10,000 rpm for 10 min, and the clear supernatant was used as the crude enzyme for determination of fibrinolytic activity on fibrin–agarose plates. The fibrin–agarose plate was made with 1% (w/v) agarose, 50 μl thrombin (100 NIH U/ml), and 0.5% (w/v) fibrinogen. These were mixed immediately and allowed to stand for 1 h at room temperature (30°C ± 2°C) to form a fibrin clot. Then 15 μl of enzyme from the bacterial isolates was dropped individually into the wells and incubated for 5 h at room temperature (30°C ± 2°C), and the lytic halo zone was measured (Astrup and Müllertz, 1952). The strain that showed the largest halo zone on the fibrin plate was selected for further studies. This selected strain was subjected to morphological and biochemical characteristics and 16S rDNA sequencing.

### Biochemical and Molecular Identification of the Selected Bacterial Isolate

The bacterial isolate with highest fibrinolytic enzyme activity was identified based on its biochemical properties (Holt et al., 1994) and 16S rDNA sequencing. The bacterial genomic DNA of the selected strain was extracted using a genomic DNA purification kit (Qiagen). The primers used were P1: 5′GAGTTTGATCMTGGCTAG3′ (upstream) and P2: 5′ACGGGCGG TGTGTRC3′ (downstream) (Rejiniemon et al., 2015). The genomic DNA was amplified by using a Polymerase Chain Reaction Peltier Thermal Cycler Machine (USA) and DNA polymerase (Sigma–Aldrich, USA). The amplified 16S rDNA PCR product was sequenced at Scigenome Laboratories, India. Further, the similarity of the sequences was checked by BLAST through the NCBI server. The 641-bp 16S rDNA sequences of the bacterial isolate were submitted to GenBank.

### Fibrinolytic Enzyme Assay

Fibrinolytic enzyme-producing capability of isolate IND7 was assayed using fibrin as a substrate. The crude enzyme was used as the sample for fibrinolytic enzyme assay. A 0.1-ml aliquot of enzyme was mixed with 2.5 ml of Tris-HCl buffer (0.1 M, pH 7.8) containing calcium chloride (0.01 M). Then, 2.5 ml fibrin (1.2%, w/v) was added and incubated for 30 min at 37°C. The enzymatic reaction was terminated by adding 5.0 ml trichloroaceticacid (0.11 M) containing sodium acetate (0.22 M) and acetic acid (0.33 M). This mixture was allowed to stand for 30 min at room temperature and centrifuged at 10,000 rpm for 10 min. The absorbance was measured with the clear supernatant at 275 nm against individual sample blank. One unit of fibrinolytic activity was defined as the amount of enzyme that liberates 1 μg of L-tyrosine per minute under the standard assay conditions.

### Substrate

The agro-wastes such as banana peel, cow dung, wheat straw, wheat bran, rice bran, rice straw, and green gram husk were collected locally and dried under sunlight for 7 days. These dried agro-wastes were powdered using a mixer grinder and used as the substrate.

### Fibrinolytic Enzyme Production under SSF

Fibrinolytic enzyme production was carried out in 100-ml Erlenmeyer flasks containing 5 g agro-wastes. The solid medium (banana peel, cow dung, wheat straw, wheat bran, rice bran, rice straw, and green gram husk) was moistened with 4 ml of Tris-HCl buffer (pH 8.0, 0.1 M). The tested substrates were sterilized individually at 121°C for 20 min, cooled, inoculated with 5% inoculum at 37°C for 48–72 h under static conditions. Enzyme was extracted by adding 50-ml double-distilled water with the fermented medium and placed in an orbital shaker at 150 rpm for 30 min at room temperature (30°C ± 2°C). It was centrifuged at 10,000 rpm for 10 min at 4°C, and the clear supernatant was used as the source of crude enzyme.

### Optimization of Fibrinolytic Enzyme Production by One-Variable-at-a-Time Approach

The process parameters were initially screened by one-variable-at-a-time approach. SSF was carried out in a 100-ml Erlenmeyer flask containing 5.0-g cow dung moistened with 5.0 ml buffer (Tris-HCl, 0.1 M, pH 8.0). The culture conditions such as the incubation period (12–96 h), pH (5.0–10.0), inoculum (2–10%), moisture content (60%–100%), carbon sources (1%, w/w; sucrose, maltose, starch, xylose, glucose, and trehalose), nitrogen sources (1%, w/w; yeast extract, casein, beef extract, peptone, urea, and gelatin), and salt solutions (0.1%, w/w, MgSO_4_, Na_2_H_2_PO_4_, ammonium chloride, sodium nitrate, calcium chloride, NaH_2_PO_4_, ferrous sulfate, and ammonium sulfate) were subjected for enzyme production. SSF was carried out as described above. Then, about 50 ml of double-distilled water was mixed with the solid medium, and fibrinolytic enzyme was extracted as described above.

### Elucidation of Process Parameters Affecting Fibrinolytic Enzyme Production Using 2^5^ Full Factorial Design

Preliminary study was carried out to evaluate the effect of tested variables on fibrinolytic enzyme production by one-variable approach. Based on these experiments, five independent variables were selected for further optimization by 2^5^ full factorial design. Two-level full factorial design was used for screening the most important nutrient and physical factors (pH) to achieve maximum fibrinolytic enzyme production by *Bacillus* sp. IND7 under SSF. Five variables were screened in 32 experimental runs. The medium components such as starch, beef extract, MgSO_4_, and physical parameters such as pH and moisture content were investigated as variables using 2^5^ full factorial design to identify the components that significantly affected fibrinolytic enzyme production. In 2^5^ full factorial design, the selected variables were examined at two levels, high (+) and low (-). Five gram of substrate was taken in Erlenmeyer flask, and the nutrient and pHs were maintained. The other factors such as inoculum and fermentation period were kept at middle level. Experiments were carried out under SSF for 72 h at 37°C. Fibrinolytic enzyme assay was carried out in triplicates, and the average value was taken as response *Y*. Analysis of variance (ANOVA) was carried out to evaluate the statistical parameters, and the “*p*” value less than 0.05 indicated that the model variables are significant. The statistical tool “Design Expert 8.0” was used for analyzing the experimental results. This experiment was based on the following first-order polynomial model:

$$\begin{array}{c} Y=\alpha_{0}+\Sigma \alpha_{i}x_{i}+\Sigma \alpha_{ij}x_{i}x_{j}+\Sigma \alpha_{ijk}x_{i}x_{j}x_{k}+\Sigma \alpha_{ijkl}x_{i}x_{j}x_{k}x_{l}+\Sigma \alpha_{ijklm}x_{i}x_{j}x_{k}x_{l}x_{m}\\ i\;ij\;ijk\;ijkl\;ijklm\; \end{array}$$

where *Y* is the response (fibrinolytic enzyme production); *α_ij_, α_ijk_, α_ijkl_*, and *α_ijklm_* were the interaction coefficients; *α*_i_ was the *i*th linear coefficient; and *α*_0_ was an intercept.

### Response Surface Methodology

The components of the medium and the interaction between the variables that significantly influenced fibrinolytic enzyme production were analyzed and optimized by central composite design (CCD). In the present study, pH of the medium (*A*) and concentrations of starch (*B*) and beef extract (*C*) were optimized by CCD. The factors were analyzed in five levels (-*α*, -1, 0, +1, and +*α*). A set of 20 experimental runs (six axial, six central, and eight factorial points) were carried out. Cow dung substrate was supplemented with different concentrations of starch and beef extract, and the pH was maintained as described in the matrix. Then, 9% (v/w) inoculum was introduced into the medium and incubated at 37°C for 72 h. The response of the variables was investigated as a function of fibrinolytic activity, as the amount of fibrinolytic enzyme produced. The experiments were conducted in triplicates, and the average enzyme activity was reported as the response (*Y*). The experiments and data were analyzed using Design Expert Version 8.0 (Stat-Ease Inc., Minneapolis, MN, USA) statistical software. The analyzed results were fitted to the second-order polynomial equation:

$$Y=\alpha_{0}+\alpha_{1}A+\alpha_{2}B+\alpha_{3}C+\alpha_{1}\alpha_{2}AB+\alpha_{1}\alpha_{3}AC+\alpha_{2}\alpha_{3}BC+\alpha_{1}\alpha_{1}A^{2}+\alpha_{2}\alpha_{2}B^{2}+\alpha_{3}\alpha_{3}C^{2},$$

where *Y* is the response (fibrinolytic activity; U/ml); *A* is the coded value of pH; *B* is the coded value of starch; *C* is the coded value of beef extract; *α*_1_, *α*_2_, and *α*_3_ are the linear coefficients; *α*_1_*α*_2_, *α*_1_*α*_3_, and *α*_2_*α*_3_ are the interactive coefficients; and *α*_1_*α*_1_, *α*_2_*α*_2_, and *α*_3_*α*_3_ are the quadratic coefficients.

To validate the theoretical model, experiments were carried out in triplicates using the optimized conditions. The theoretical values were compared with the observed results obtained in triplicate experiments and validated.

### Determination of Protein Concentration

Total protein was measured using bovine serum albumin as the standard (Lowry et al., 1951).

### Sodium Dodecyl Sulfate Polyacrylamide Gel Electrophoresis (SDS-PAGE) and Zymogram Analysis

Sodium dodecyl sulfate polyacrylamide gel electrophoresis was performed using 12% polyacrylamide gel (Laemmli, 1970). Twenty micro gram protein sample was loaded on SDS-PAGE to determine the molecular weight of proteins from the extracellular lysate from *Bacillus* sp. IND7. Two ranges of protein markers (205 kDa – 29 kDa and 43 kDa – 14 kDa) were applied to determine the molecular weight of proteins. Fibrin zymography was carried out to determine fibrinolytic activity of the enzyme on SDS-PAGE. The SDS-PAGE (12%) was copolymerized with 0.2% fibrinogen and 50 μl thrombin (10 NIH units). The crude sample was unheated and the loaded SDS-sample buffer lacking reducing agent. After enzyme separation, the gel was soaked in 2.5% Triton X-100 for 30 min at 30°C ± 2°C and further washed with double-distilled water. This gel was further incubated in 0.1 M sodium phosphate buffer (pH 7.4, 0.1 M) for 5 h at 30°C ± 2°C. The gel was stained with CBB R-250 for 1 h and then destained. The molecular mass of the fibrinolytic enzyme was evaluated with the protein marker, namely phosphorylase b (97.4 kDa), bovine serum albumin (66 kDa), ovalbumin (43 kDa), carbonic anhydrase (29 kDa), and soybean trypsin inhibitor (20.1 kDa).

### *In Vitro* Blood Clot Lytic Activity of Fibrinolytic Enzyme

About 0.5-ml aliquots of goat blood were collected from the slaughter house at Nagercoil, India and allowed to form a clot. The clot was washed thrice with phosphate-buffered saline and incubated with crude fibrinolytic enzyme (120 μg). Streptokinase was used as a positive control, and phosphate-buffered saline was used as the negative control. All tubes were incubated at room temperature (30°C ± 2°C) for 6 h, and the results were observed for every 30 min.

## Result

### Bacterial Strain

**FIGURE 1:**
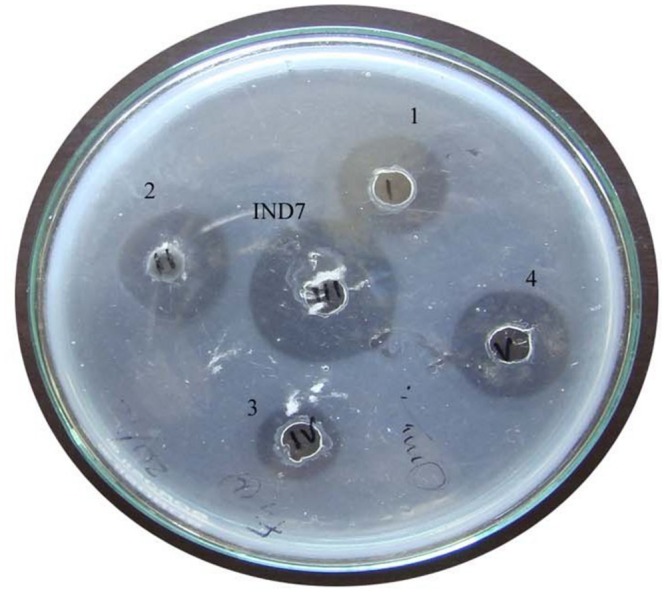
**Fibrinolytic activity of enzyme from the bacterial isolates**.

Totally, 34 morphologically dissimilar protease positive bacterial isolates were obtained. Among the 34 isolates, five potent isolates were chosen for secondary screening. The isolate *Bacillus* sp. IND7 displayed strong fibrinolytic activity. This organism produced 8-mm zone on fibrin plate (**Figure 1**). The fibrinolytic activity of *Bacillus* sp. IND7 was relatively higher than the selected bacterial isolates. The identified strain was Gram positive, motile, rod shaped, catalase- and casein positive, and starch hydrolyzing. Whereas, the strain revealed negative results toward indole test, urea hydrolysis, citrate test, and nitrate reduction. The strain IND7 was identified as *Bacillus* sp. IND7, and 641-bp sequences were submitted under the accession number KF250422.

### Evaluation of Agroresidues

**FIGURE 2:**
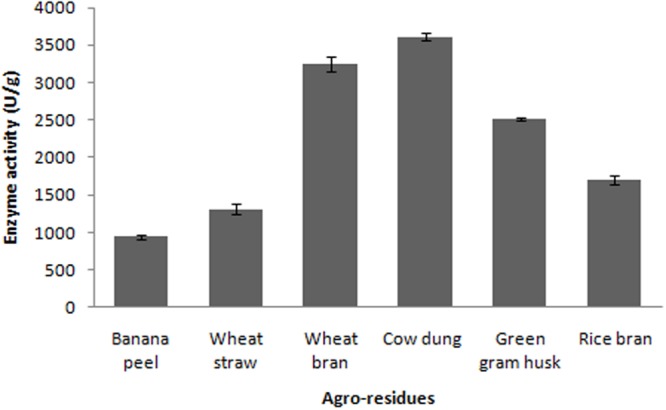
**Screening of agroindustrial wastes for the production of fibrinolytic enzyme**.

In the present study, various agroresidues were screened for maximum production of fibrinolytic enzymes. The present results revealed that fibrinolytic enzyme production by *Bacillus* sp. IND7 depends on the substrate used in SSF. Maximum fibrinolytic enzyme production was observed with cow dung substrate (3,613 ± 49.2 U/g). Enzyme production was 941 ± 32.4, 1,312 ± 68.3, 3,247 ± 102.6, 1,698 ± 63.2, and 2,513 ± 26.9 U/g, respectively, for banana peel, wheat straw, wheat bran, rice bran, and green gram husk, respectively (**Figure 2**). The fibrinolytic enzyme production by the selected bacteria on various agroindustrial residues is described in **Table 1**.

**Table 1:** Fibrinolytic enzyme production on various agroindustrial residues in solid-state fermentation from bacterial isolates.

Microorganisms	Substrate	Reference
*Bacillus subtilis*	Red bean	Chang et al., 2012
*Bacillus altitudinis* GVC11	Castor husk	Madhuri et al., 2012
*Bacillus cereus* IND1	Wheat bran	Vijayaraghavan and Vincent, 2014
*Virgibacillus* sp. SK 37	Brewery yeast sludge	Lapsongphon et al., 2013
*Bacillus amyloliquefaciens* FCF-11	Corn husk	Kotb, 2014
*Bacillus amyloliquefaciens*	Chick peas	Wei et al., 2011
*Bacillus subtilis*	Soybean curd residues	Zu et al., 2010
*Bacillus subtilis* I-2	Soybean meal	Bajaj et al., 2014
*Bacillus* sp. IND7	Cow dung	Present study

### Optimization of Enzyme Production by Traditional Method

**FIGURE 3:**
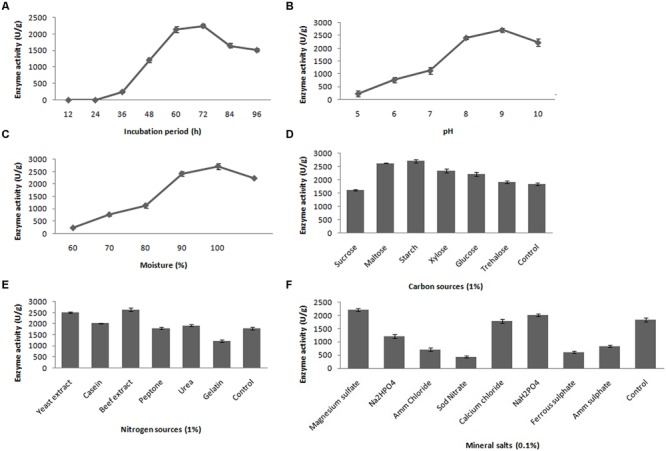
**Effect of incubation time **(A)**, pH **(B)**, moisture **(C)**, carbon source **(D)**, nitrogen source **(E)** and mineral salts **(F)** on fibrinolytic enzyme production.** *Error bar* represents standard deviation.

The fibrinolytic activity of the selected stain was subjected at various incubation periods (12–96 h); however, maximum fibrinolytic activity (2417.8 ± 61.6 U/g) was observed after 72-h incubation at 37°C (**Figure 3A**). In the further optimization steps, the fermentation period was maintained as 72 h. To determine the effect of pH for fibrinolytic enzyme production, the fermented medium was inoculated and incubated in pH ranging from 5.0 to 10.0. The maximum fibrinolytic activity was recorded at pH 9.0 (2717.4 ± 79.2 U/g) (**Figure 3B**). Further increase of pH depleted enzyme activity. The pH of the culture medium was maintained as 9.0 for further experiments. In SSF, moisture content is one of the critical factors. In the present study, the optimum moisture content of the medium was evaluated for enhanced production of fibrinolytic enzyme. The minimum fibrinolytic activity (148.6 ± 13.1 U/g) was observed with lower moisture content (60%), while the maximum (2441.2 ± 102.4 U/g) was observed at 90% moisture content (**Figure 3C**). Further increase in initial moisture content of the culture medium caused decrease in fibrinolytic enzyme activity.

Different carbon sources were evaluated to determine their effects on fibrinolytic activity of *Bacillus* sp. IND7. The results revealed that this organism could utilize a range of carbon source, and maximum fibrinolytic activity was observed with the culture medium supplemented with starch (2713.8 ± 64.9 U/g). Enhanced fibrinolytic activity (1912.7 ± 49.4 – 2627.4 ± 17.8 U/g) was observed with other carbon sources such as trehalose, xylose, glucose, and maltose (**Figure 3D**). Different nitrogen sources were examined to elucidate their effects on fibrinolytic enzyme production. The results showed that the selected bacterial strain utilized almost all tested nitrogen sources. Maximum fibrinolytic enzyme production was observed with the culture medium supplemented with beef extract (2645.6 ± 68.5 U/g) (**Figure 3E**). Different mineral salt solutions were examined to determine their effects on fibrinolytic activity of the isolate (**Figure 3F**). Maximum fibrinolytic enzyme production was observed with magnesium sulfate (2217.8 ± 39.4 U/g). The ions such as ammonium chloride and ferrous sulfate negatively influenced fibrinolytic enzyme production.

### Screening of Variables by Statistical Approach

In the present study, the nutrient factors such as starch (carbon source), beef extract (nitrogen source), and MgSO_4_ (mineral salt) were selected after the preliminary experiments by one-variable-at-a-time approach. A two-level full factorial design was used to evaluate the maximum fibrinolytic enzyme production as a function of moisture, starch, beef extract, MgSO_4_, and pH. The variables and their levels were described in **Table 2A**. The experimental design and results of 2^5^ full factorial design were described in **Table 2B**. In this model, the fibrinolytic enzyme production varied from 2,480 to 7,510 U/g in the 32 trials. Fibrinolytic enzyme production was maximum in the medium containing 1% starch, 0.05% beef extract, 0.01% MgSO_4_, with pH 9.0. The “*F*” value of the model was 17.51, and this model is statistically significant (*p* < 0.0001). There is only a 0.01% chance that a “Model *F*-value” this large could occur due to noise. The tested variables such as pH, moisture, and beef extract were statistically significant (*p* < 0.05). The other factors such as moisture and MgSO_4_ did not affect significantly on enzyme production (*p* > 0.05) (**Table 2C**.). The effect estimate was positive to pH, moisture, maltose, and beef extract, while the effect estimate was negative to MgSO_4_. The interactive effects such as AB, AD, BC, BD, BE, CE, DE, ABC, ACD, ADE, BCE, ABCD, ABCE, ACDE, and BCDE were statistically significant (*p* < 0.05). The *R*^2^ of this model was 0.9696, and the adjusted *R*^2^ was 0.9142. In this model, the adequate precision measures the signal-to-noise ratio, and the ratio greater than 4 is desirable. In this model, the adequate precision obtained was 20.885, which indicated adequate model. Neglecting the insignificant variables, the model equation for fibrinolytic enzyme production can be written as follows:

**Table 2A:** Factors and their levels of independent variables.

Variables	Name	Units	Coded levels	
			–1	+ 1
A	pH		7.0	9.0
B	Moisture	%	80	100
C	Starch	%	0.1	1.0
D	Beef extract	%	0.05	0.5
E	MgSO_4_	%	0.01	0.1

**Table 2B:** Two-level full factorial matrix for screening of variables for fibrinolytic enzyme production from *Bacillus* sp. IND7.

Run	pH	Moisture	Starch	Beef extract	MgSO_4_	Enzyme activity
	(A)	(B)	(C)	(D)	(E)	(U/g)
1	7	80	1	0.5	0.1	4720
2	9	100	0.1	0.05	0.01	5205
3	7	80	1	0.05	0.01	5205
4	7	80	0.1	0.05	0.1	5740
5	9	80	1	0.05	0.01	7510
6	7	80	0.1	0.5	0.1	5700
7	7	80	1	0.05	0.1	6740
8	9	100	0.1	0.5	0.01	6430
9	9	80	0.1	0.05	0.01	4030
10	9	80	0.1	0.5	0.01	5505
11	9	80	0.1	0.05	0.1	4665
12	9	100	1	0.5	0.1	5630
13	9	80	0.1	0.5	0.1	2480
14	7	100	0.1	0.5	0.01	5505
15	9	100	0.1	0.5	0.1	6325
16	7	80	0.1	0.05	0.01	5255
17	7	100	1	0.05	0.1	6985
18	9	100	1	0.5	0.01	6375
19	7	100	1	0.5	0.1	6930
20	9	80	1	0.5	0.1	6260
21	7	80	0.1	0.5	0.01	5955
22	9	100	1	0.05	0.01	4590
23	9	100	0.1	0.05	0.1	6235
24	9	80	1	0.05	0.1	5290
25	7	100	0.1	0.05	0.1	3805
26	7	100	1	0.5	0.01	6065
27	9	100	1	0.05	0.1	6020
28	7	100	0.1	0.05	0.01	6135
29	7	100	1	0.05	0.01	6150
30	7	100	0.1	0.5	0.1	4515
31	7	80	1	0.5	0.01	6645
32	9	80	1	0.5	0.01	6670

**Table 2C:** Analysis of variance for two-level full factorial design for the production of fibrinolytic enzyme.

Source	Sum of Squares	df	Mean Square	*F* Value	*p*-value
Model	3.545E + 07	20	1.7730E + 06	17.51	<0.0001
A-pH	1.086E + 06	1	1.0860E + 06	10.73	0.0074
B-Moisture	2.7840E + 05	1	2.7840E + 05	2.75	0.1255
C-Starch	5.0840E + 06	1	5.0840E + 06	50.21	<0.0001
D-Beef extract	4.2670E + 05	1	4.2670E + 05	4.21	0.0547
E-MgSO_4_	2.3380E + 05	1	2.3380E + 05	2.31	0.1569
AB	4.0150E + 06	1	4.0150E + 06	39.65	<0.0001
AD	5.68E + 05	1	5.68E + 05	5.61	0.0372
BC	1.79E + 06	1	1.79E + 06	17.71	0.0015
BD	9.82E + 05	1	9.82E + 06	9.7	0.0099
BE	1.41E + 06	1	1.41E + 06	13.92	0.0033
CE	9.33E + 05	1	9.33E + 05	9.22	0.0113
DE	2.84E + 06	1	2.81E + 06	28.06	0.0003
ABC	3.12E + 06	1	3.12E + 06	30.77	0.0003
ACD	9.27E + 05	1	9.27E + 05	9.15	0.0116
ADE	6.29E + 05	1	6.29E + 05	6.21	0.0299
BCE	2.05E + 06	1	2.05E + 06	20.22	0.0009
ABCD	2.45E + 06	1	2.45E + 06	24.15	0.0005
ABCE	4.35E + 06	1	4.35E + 06	42.94	<0.0001
ACDE	9.10E + 05	1	9.10E + 05	8.98	0.0121
BCDE	1.38E + 06	1	1.38E + 06	13.63	0.0036
Residual	1.11E + 06	11	1.01E + 05		
Cor Total	3.66E + 07	31			

$$\begin{array}{c} \mathrm{Enzyme}\;\mathrm{activity}=\;\;\;\;\;\;\;\;\;\;\;\;\;\;\;\;\;\;\\ \;+5587.97+184.22A+93.28B+398.59C+115.47D\\ -85.47E+354.22AB+133.28AD-236.72BC\;\\ \begin{array}{c} \;+175.16BD+209.84BE+170.78CE-140.16ADE\\ \begin{array}{c} +252.97BCE-276.41ABCD-368.59ABCE\;\;\\ +168.59ACDE-207.66BCDE.\;\;\;\;\;\;\; \end{array} \end{array} \end{array}$$

The two nutrient factors (starch and beef extract) and pH were further investigated with CCD. The other factors such as moisture (%) and MgSO_4_ were kept at middle level.

### Response Surface Methodology

Central composite design was applied to study the interaction among the selected medium components and to determine their optimum levels for enhanced fibrinolytic enzyme production. The factors and their levels for CCD design are described in **Table 3A**. The variables used for the 2^3^ full factorial design analysis were pH (*A*), starch (*B*), and beef extract (*C*). The statistical combination of the media components along with observed and predicted responses (fibrinolytic enzyme activity) is presented in **Table 3B**. In this model, the fibrinolytic enzyme production was maximum in the medium containing 1% starch and 0.5% beef extract with medium pH 9.68. The statistical significance of the second-order polynomial model equation was analyzed by *F*-test and was described in **Table 3C**. The statistical analysis yielded a regression equation shows the empirical relationship of variables. The model *F*-value of 94.65 implied that this model was statistically significant. The “lack-of-fit *F*-value” of this model was 0.02, which implied that the lack of fit of this model was non-significant. Non-significant lack of fit is good. The “predicted *R*^2^” of this model was 0.9919 and was reasonable agreement with the “adjusted *R*^2^” of 0.9780. Adequate precision measures the signal-to-noise ratio and the value >4 is desirable. In this model, the ratio of 35.085 indicated adequate signal. The coefficient of variation of this model was 6.37, which denotes that the performed experiment is highly reliable. The optimum concentration of selected variables was obtained by analyzing the response surface plots resolving the regression equation. The regression equation obtained after the ANOVA provides an estimate of the level of fibrinolytic enzyme production as a function of pH, starch, and beef extract. The developed experimental design for predicting fibrinolytic enzyme activity of IND7 was found to be more accurate in optimizing the closed medium components:

**Table 3A:** Independent variables and their ranges used in response surface methodology.

Variables	Symbols			Coded values		
		
		–α	–1	0	+1	+α
pH	A	6.32	7.0	8.0	9.0	9.68
Starch	B	0.16	0.5	1.0	1.5	1.84
Beef extract	C	0.08	0.25	0.5	0.75	0.92

**Table 3B:** Actual and predicted values of fibrinolytic enzyme observed in experimental setup of response surface methodology.

Run	pH (A)	Starch (B)	Beef extract (C)	Enzyme activity	
				Observed	Predicted
1	9.68	1	0.5	8278	8276
2	8	1	0.5	6234	6548
3	8	0.16	0.5	4756	4798
4	8	1	0.5	6748	6548
5	8	1	0.92	5799	5828
6	8	1	0.5	6711	6548
7	8	1	0.08	6410	6407
8	9	1.5	0.25	8154	8166
9	8	1	0.5	6691	6548
10	8	1	0.5	6698	6548
11	9	0.5	0.75	6020	5594
12	7	0.5	0.25	4921	4895
13	8	1.84	0.5	4746	4730
14	9	0.5	0.25	6417	6411
15	6.32	1	0.5	4482	4510
16	9	1.5	0.75	6744	6749
17	8	1	0.5	6212	6549
18	7	1.5	0.75	3801	3787
19	7	0.5	0.75	5654	5622
20	7	1.5	0.25	4052	4058

**Table 3C:** Analysis of variance for response surface quadratic model of fibrinolytic enzyme production from *Bacillus* sp. IND7.

Source	Sum of squares	df	Mean square	*F* Value	*p*-value
Model	2.789E + 07	9	3.9900E + 06	94.65	<0.0001
A-pH	1.712E + 07	1	1.7120E + 07	522.82	<0.0001
B-Starch	5.6516E + 03	1	56.51.58	0.17	0.6866
C-Beef extract	4.0530E + 05	1	4.0530E + 05	12.38	0.0056
AB	3.3580E + 06	1	3.3580E + 06	102.54	<0.0001
AC	6.55E + 05	1	6.55E + 05	20	0.0012
BC	4.9850E + 05	1	4.9850E + 05	15.22	0.003
A^2^	4.3108E + 04	1	4.3108E + 04	1.32	0.278
B^2^	5.73E + 06	1	5.73E + 06	175.02	<0.0001
C^2^	3.33E + 05	1	3.33E + 05	10.18	0.0096
Residual	3.28E + 05	10	3.27E + 04		
Lack of Fit	6.47E + 03	5	1293.69	0.02	0.9997
Pure Error	3.21E + 05	5	6.42E + 04		
Cor Total	2.82E + 07	19			

$$\begin{array}{c} \mathrm{Enzyme}\;\mathrm{activity}=\;\;\;\;\;\;\;\;\;\;\;\;\;\;\;\;\;\;\;\;\\ +6548.23+1119.66A-20.34B-172.26C+647.88AB\;\;\\ -286.12AC-249.62BC-54.69A^{2}-630.63B^{2}-152.10C^{2}. \end{array}$$

**FIGURE 4:**
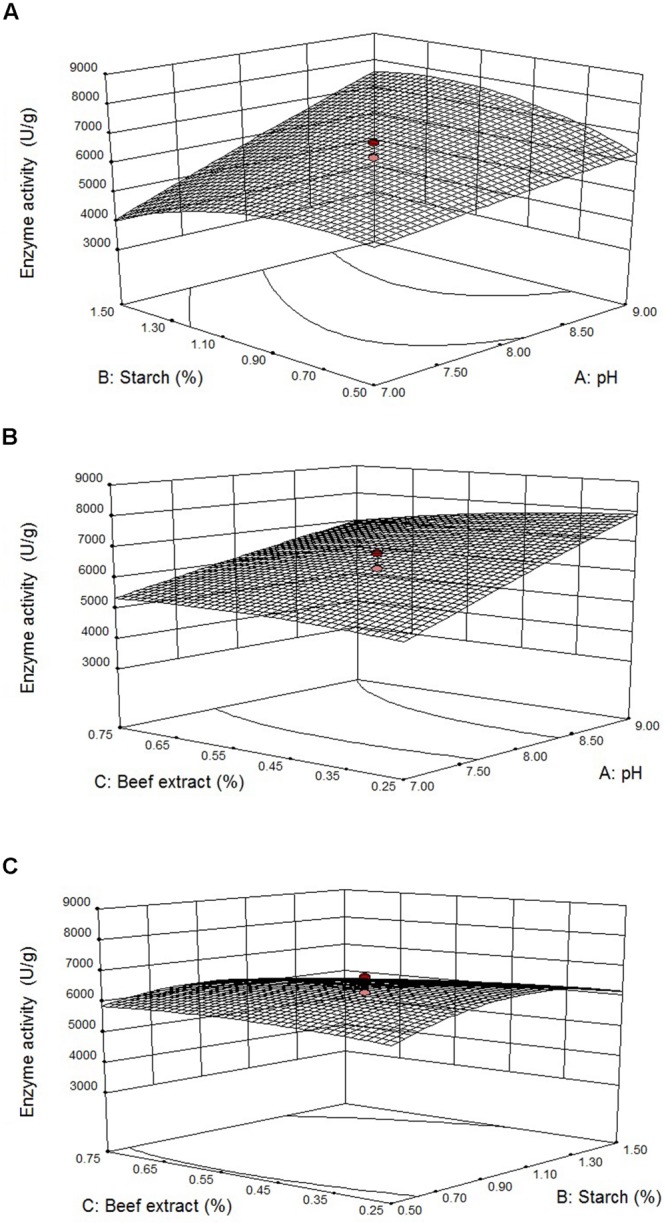
**Effect of pH vs. starch **(A)**, pH vs. beef extract **(B)**, and starch vs beef extract **(C)** on fibrinolytic enzyme production.** *Error bar* represents standard deviation.

**Figures 4A–C** illustrates three-dimensional (3D) plot of pH, starch, and beef extract on the fibrinolytic enzyme production. It is evidence that the fibrinolytic enzyme yield increased when pH of the medium increased. The interactive effect of factors on fibrinolytic enzyme yield was studied by changing the level of any one independent variable while keeping the other independent variables at its constant level. The 3D plots clearly showed that the maximum fibrinolytic enzyme activity occurs at higher pH and starch and lowest concentration of beef extract and MgSO_4_.

Results showed that starch enhanced enzyme production upto 1.23%, and further increase of its concentration declined enzyme production. Lowest concentration of beef extract (0.3%) preferred for fibrinolytic enzyme production, and higher concentration negatively influenced enzyme production. According to the point prediction analysis using response surface plots, medium starch concentration of 1.23% (w/w), beef extract 0.3% (w/w), and pH 9.0 significantly enhanced fibrinolytic enzyme production. After statistical optimization, the enzyme production of fibrinolytic activity was about 2.5-fold.

### Validation of the Quadratic Model

Validation of the optimized media composition was done using SSF with predicted optimum values of different parameter. The optimum medium composition and process parameters for fibrinolytic enzyme production were 1.23% starch, 0.3% beef extract, and pH 9.0. Under these experimental conditions, the fibrinolytic enzyme yield was found to be 8,345 U/g, which was excellent agreement with the predicted response of 8,263 U/g. The model developed in the present study was accurate and reliable for predicting the production of fibrinolytic enzyme in SSF from *Bacillus* sp. IND7.

**FIGURE 5:**
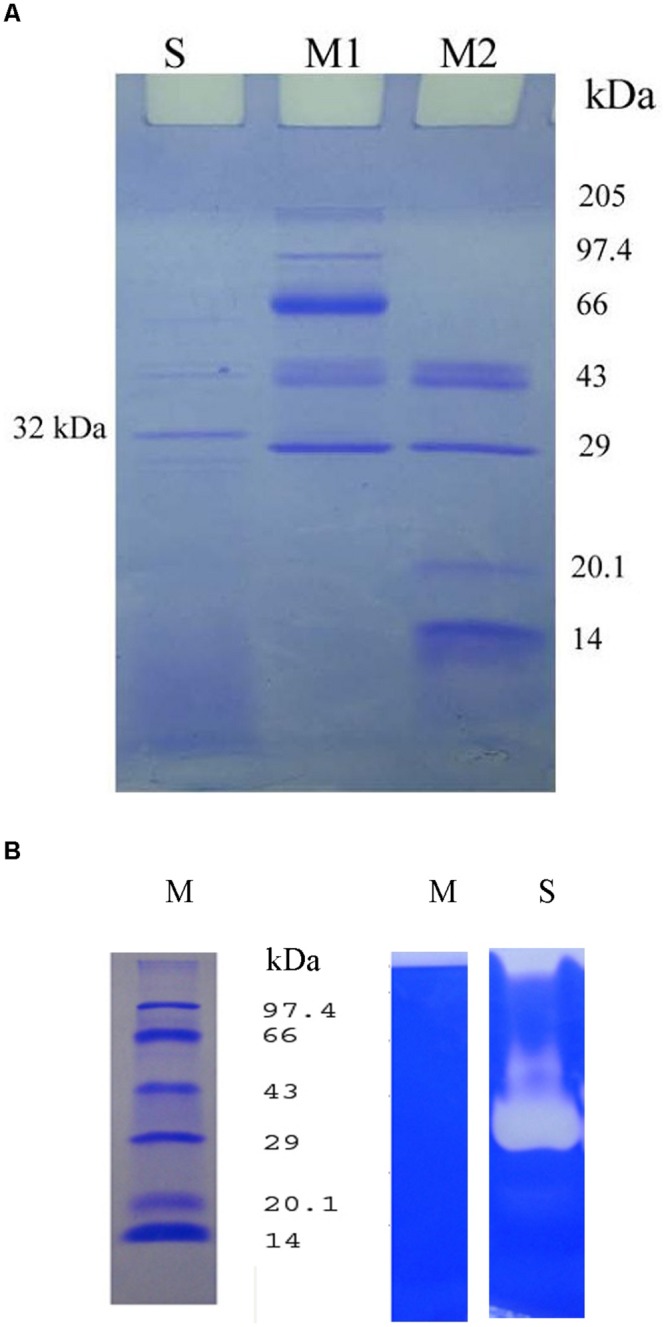
**(A)** Electrophoretic analysis of the crude enzyme from *Bacillus* sp. IND7 (S- Crude fibrinolytic enzyme; M1-Protein marker [205 kDa – 29 kDa]; M2 – Protein marker [43 kDa-14 kDa]). **(B)** Zymogram analysis of the crude enzyme on 12% (w/v) sodium dodecyl sulfate – polyacrylamide slab gel (M, Protein marker; S, Crude fibrinolytic enzyme). Two ranges of protein markers (205 kDa – 29 kDa and 43 kDa – 14 kDa) were applied to determine the molecular weight of proteins.

SDS-PAGE of the crude extracellular lysate of *Bacillus* sp. IND7 revealed a major protein band with molecular mass of approximately 32 kDa (**Figure 5A**). The fibrinolytic enzyme activity of the crude sample was determined in 12% SDS-PAGE. The fibrinolytic enzyme of *Bacillus* sp. IND7 appeared as a colorless zone on this gel (**Figure 5B**). The blood clot lytic activity of fibrinolytic enzyme from *B. thuringiensis* IND7 was evaluated in *in vitro*. The crude enzyme digested blood clot completely within 6 h at room temperature (30 ± 2°C).

## Discussion

In this study, a fibrinolytic enzyme-producing bacterium was screened and isolated from cooked rice sample. The strain IND7 showed excellent zone on fibrin–agarose plate. The biochemical properties and 16S rDNA sequence was analyzed, and the strain was designated as *Bacillus* sp. IND7. Fibrinolytic enzyme screening from various sources, such as Japanese *shiokara* (Sumi et al., 1995), Indonesian *tempeh* (Kim et al., 2006), and fermented red bean (Chang et al., 2012), has been carried out. The bacterial fibrinolytic enzymes are considered as the safe thrombolytic agent, and the administration of these fibrinolytic agents upon oral administration could increase fibrinolytic activity in human plasma (Sumi et al., 1990). Hence, the studies on bacterial fibrinolytic enzyme, especially from the genus *Bacillus*, could be useful to develop potent thrombolytic agents.

Enzymes can be produced by submerged fermentation (SmF) and SSF. SSF has many advantages over SmF (Pandey et al., 2000). Hence, in this study, SSF was performed for the production of fibrinolytic enzyme. In SSF, many substrates have used for the production of fibrinolytic enzyme. Comparing the reported agroresidues, cow dung substrate is cheap. The main criteria for the selection of an ideal substrate for SSF are cost, availability of the substrates (Pandey et al., 2000), and nutrient composition of the selected solid waste (Vijayaraghavan et al., 2015). Cow dung medium may be considered as a promise and in expensive substrate for fibrinolytic enzyme production. Cow dung contains high amounts of nutrients (Misra et al., 2003). It is rich in total organic carbon (42.5%), nitrogen (0.65%), phosphorus (0.5%), potassium (0.125%), sodium (0.138%), calcium, iron, copper, zinc and cadmium (Yadav et al., 2013). So, the bacteria can be grown on this medium for the production of fibrinolytic enzymes. Banana peel is rich of organic matter (91.5 ± 0.05%), carbohydrate (59 ± 1.36%), crude fiber (31.7 ± 0.25%), crude lipid (1.7 ± 0.1) and low amount of crude protein (0.9 ± 0.25%) (Anhwange et al., 2009). Rice straw contains less amount of protein (0.469%), fiber (3.289%), cellulose (4.374%), hemicelluloses (1.368%) and traces of sodium, potassium, calcium, phosphorus, and magnesium (Akinfemi and Ogunwole, 2012). Wheat straw is rich in cellulose (33.7%), hemicellulose (21%), lignin (11%), fiber (54%), protein (3.6%), calcium (0.18%) and phosphorus (0.05%) (Yasin et al., 2010). Rice bran contains more quantity of protein (16.16%), total lipids (17.87%), carbohydrates (33.24%) and traces of calcium, iron, sodium, zinc, and potassium (Faria et al., 2012). Green gram husk contains carbohydrate (60.52%), crude protein (7.69%), crude fat (2.17%), crude fiber (18.63%), iron, calcium, phosphorus, zinc, copper, and manganese (Bora and Kulshrestha, 2014). Considering the cheap cost and its availability, cow dung could spearhead enzyme bioprocess in an industrial scale. Reports on fibrinolytic enzyme production using cow dung substrate in SSF from *Bacillus* sp. are limited or perhaps not available. This is the first report on the production of fibrinolytic enzyme from *Bacillus* sp. IND7 using cow dung substrate in SSF. Hence, the present study is useful to utilize the cow dung substrate for the production of fibrinolytic enzyme by *Bacillus* sp. The agroresidues such as rice bran and wheat bran are considered as waste, but these substrates are useful to make feed for aquatic organisms and poultry (Vijayaraghavan and Vincent, 2012). In this experiment 80% moisture content was maintained for the production of fibrinolytic enzyme. In SSF, moisture content is one of the critical factors. Cow dung substrate has high moisture-holding capacity, which facilitates the production of fibrinolytic enzyme from bacterial species.

Optimization of pHs and nutrient content in any bioprocess is primarily important to develop efficient process. Initially, the pH of the medium and the nutrient components were optimized by one-variable-at-a-time approach. Fibrinolytic enzyme production was maximum after 72 h of incubation, at pH 9.0, and with 90% moisture level. The fermentation period requirement of the strain IND7 for the maximum fibrinolytic activity is comparable with that of different fibrinolytic enzyme-producing bacteria. In the present study, optimum pH for enhanced production of fibrinolytic enzyme was found to be 9.0, and fibrinolytic activity was 2717.2 ± 79.2 U/g. Hence, the pH of culture medium was maintained as 8.0 for further studies. In SSF, moisture is one of the critical factors that strongly influence the enzyme yield. The optimum moisture content for enzyme production could vary depend on the organism and substrate used in SSF process (Prakasham et al., 2006). In the present study, various carbon and nitrogen sources were used for enhanced production of fibrinolytic enzyme. Results revealed that starch favors maximum fibrinolytic enzyme production, followed by sucrose. This result was in agreement with the previous study on proteolytic enzymes from Bacillus sp. (Sepahy and Jabalameli, 2011). In the present study, beef extract enhanced maximum enzyme production. Similar positive effects of beef extract has also been reported in B. cereus IND1 (Vijayaraghavan and Vincent, 2014). In the present study, among six tested mineral salts, maximum fibrinolytic enzyme production was observed in the presence of magnesium sulfate at 0.1% concentration. Therefore, further optimization studies were carried out using MgSO_4_ as a mineral salt. It was also registered in previous studies where MgSO_4_ has been found to be suitable inducer for the production of fibrinolytic enzyme by B. subtilis BS-26 (Niu et al., 2008).

The traditional one-variable-at-a-time approach is simple, but it fails to explain the interaction between the variables (He et al., 2004). Hence, statistical methods of optimization have been introduced to enhance the production of fibrinolytic enzyme. Statistical approaches have been used to screen the variables and elucidation of optimum response of the variables (Box and Behnken, 1960). Factorial experimental design is one such statistical method to study the most important component in the fermentation medium formula in enzyme bioprocess. The statistical methods such as Plackett–Burman (Mukherjee and Rai, 2011), two-level fractional factorial design (Liu et al., 2005), and two-level full factorial design (Vijayaraghavan and Vincent, 2014) were used for the production of fibrinolytic enzyme from *Bacillus* sp. strain AS-S20-1, *B. natto* NLSSE, and *B. cereus* IND1, respectively. In the present study, using two-level full factorial design, a total of five variables were analyzed in 32 experimental runs. The “*F*” value of this model was 17.51, and the “*p*” value was <0.0001. Among the factors screened by statistical methods, initial pH of the medium significantly influenced the production of fibrinolytic enzyme. The three most significant factors that significantly influence the production of fibrinolytic enzyme were selected for optimization studies with RSM.

The concentrations of the media composition have adverse effect on fibrinolytic enzyme production. Therefore, optimization of individual medium components has been evaluated as a measure to decrease the production cost of fibrinolytic enzyme by microorganisms. RSM is a well-known statistical method that employs the cost-effective experimental design and has advantages such as predicted response and evaluation. In the present study, RSM was applied with CCD to optimize media components, namely pH, starch, and beef extract to improve the fibrinolytic enzyme production by *Bacillus* sp. IND7. In an enzyme bioprocess, optimization for enhanced production is great challenge to develop feasible methods (Hao et al., 2006). Statistical optimization of fibrinolytic enzyme production was attempted by various researchers (Liu et al., 2005; Deepak et al., 2008; Mukherjee and Rai, 2011; Vijayaraghavan and Vincent, 2015). RSM had been successfully employed for the enhanced production of chitin deacetylase (Pareek et al., 2014), alkaline protease (Puri et al., 2002), L-glutaminase (Singh et al., 2013), lipase (Ruchi et al., 2008), esterase (Ren et al., 2006), and keratinase (Paul et al., 2014). However, very little studies were reported to use RSM to optimize the fibrinolytic enzyme production in SSF. To the best of our knowledge, this is the first study that deals the optimization of medium components for the production of fibrinolytic enzyme from *Bacillus* sp. in SSF by statistical method. The *R*^2^ of this model was 0.9919, which shows that this model was very effective in optimizing the selected individual factors. The *R*^2^ value could be at least 0.8 to fit any model design (Yuguo et al., 1999), and the present study showed good fit with this model design.

The 3D plots allow direct visualization of interactive effect of variables and individual factors on enzyme production (Singh et al., 2014). In the present study, 3D plots showed that the maximum fibrinolytic enzyme production was obtained with high pH value, starch, and lowest value of beef extract. Interestingly, initial pH of the medium significantly influenced enzyme production than the selected supplements such as starch and beef extract. These studies imply that cow dung contains significant amount of carbon and nitrogen sources for the production of fibrinolytic enzyme from *Bacillus* sp. The RSM-optimized culture medium enhanced 2.5-fold enzyme production than unoptimized medium. The optimization of fibrinolytic enzyme production by *Bacillus* sp. IND7 of 8345 U/g achieved was higher than the optimized fibrinolytic enzyme production achieved in other works in SSF which employed *B. subtilis* (104.9 U/g) (Jung and Lee, 2009), *B. subtilis* HA (30 U/g) (Kim and Lee, 2009), *B. amyloliquefaciens* (39.28 U/g) (Wei et al., 2011) and *B. subtilis natto* (2503.4 U/g) (Nie et al., 2015), demonstrating that cow dung is an ideal culture medium for fibrinolytic enzyme production from *Bacillus* sp. IND7.

SDS-PAGE of the crude extracellular lysate revealed the protein pattern of *Bacillus* sp. IND7. The fibrinolytic enzyme appeared as a colorless zone on zymography gel. The molecular weight of the enzyme was approximately estimated as 32 kDa. Results revealed that this organism synthesized one fibrinolytic enzyme which was highly correlated with a major protein separated on 12% SDS-PAGE. The molecular weight of the fibrinolytic enzyme from *Bacillus* sp. IND7 was comparable with fibrinolytic enzymes from *B. subtilis* KCK-7 (Paik et al., 2004) and *Bacillus* sp. KDO-13 (Lee et al., 2001). The crude enzyme of *Bacillus* sp. IND7 digested blood clot directly, and this kind of *in vitro* blood clot lytic studies was recorded previously with various organisms (Deepak et al., 2008; Mahajan et al., 2012; Yuan et al., 2012; Vijayaraghavan and Vincent, 2014).

## Conclusion

In the present study, the enhanced production of fibrinolytic enzyme from *Bacillus* sp. IND7 was achieved using cow dung under SSF. The interactions of pH, starch, and beef extract were investigated by RSM. The enzyme production was found to be significantly influenced by pH of the solid medium than the feed supplements such as starch and beef extract. This clearly implies that enhanced production of fibrinolytic enzyme can be achieved by simply adjusting the pH of the cow dung substrate. These kinds of studies help to utilize cow dung substrate in enzyme bioprocess. *In vitro* clot lysis revealed its activity on blood clot.

## Author Contributions

All authors listed, have made substantial, direct and intellectual contribution to the work, and approved it for publication.

## Conflict of Interest Statement

The authors declare that the research was conducted in the absence of any commercial or financial relationships that could be construed as a potential conflict of interest.
